# Multiomics Point of Departure (moPOD) Modeling Supports an Adverse Outcome Pathway Network for Ionizing Radiation

**DOI:** 10.1021/acs.est.2c04917

**Published:** 2023-02-17

**Authors:** You Song, Keke Zheng, Dag Anders Brede, Tânia Gomes, Li Xie, Yetneberk Kassaye, Brit Salbu, Knut Erik Tollefsen

**Affiliations:** † Norwegian Institute for Water Research (NIVA), Økernveien 94, 0579 Oslo, Norway; ‡ Centre for Environmental Radioactivity (CERAD), Norwegian University of Life Sciences (NMBU), Post box 5003, N-1432 Ås, Norway; § Faculty of Environmental Sciences and Natural Resource Management (MINA), Norwegian University of Life Sciences (NMBU), Post box 5003, N-1432 Ås, Norway

**Keywords:** γ radiation, *Daphnia*, multiomics, benchmark dose modeling (BMD), adverse outcome pathway (AOP), weight of evidence

## Abstract

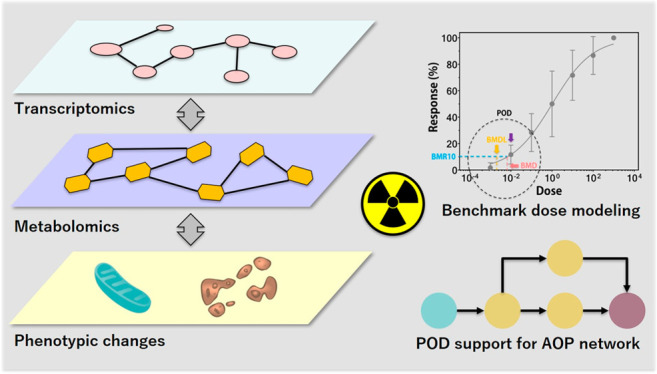

While adverse biological effects of acute high-dose ionizing radiation have been extensively investigated, knowledge on chronic low-dose effects is scarce. The aims of the present study were to identify hazards of low-dose ionizing radiation to *Daphnia magna* using multiomics dose–response modeling and to demonstrate the use of omics data to support an adverse outcome pathway (AOP) network development for ionizing radiation. Neonatal *D. magna* were exposed to γ radiation for 8 days. Transcriptomic analysis was performed after 4 and 8 days of exposure, whereas metabolomics and confirmative bioassays to support the omics analyses were conducted after 8 days of exposure. Benchmark doses (BMDs, 10% benchmark response) as points of departure (PODs) were estimated for both dose-responsive genes/metabolites and the enriched KEGG pathways. Relevant pathways derived using the BMD modeling and additional functional end points measured by the bioassays were overlaid with a previously published AOP network. The results showed that several molecular pathways were highly relevant to the known modes of action of γ radiation, including oxidative stress, DNA damage, mitochondrial dysfunction, protein degradation, and apoptosis. The functional assays showed increased oxidative stress and decreased mitochondrial membrane potential and ATP pool. Ranking of PODs at the pathway and functional levels showed that oxidative damage related functions had relatively low PODs, followed by DNA damage, energy metabolism, and apoptosis. These were supportive of causal events in the proposed AOP network. This approach yielded promising results and can potentially provide additional empirical evidence to support further AOP development for ionizing radiation.

## Abstract 2

This study describes a computational new approach methodology to assess the environmental hazards of ionizing radiation to aquatic organisms.

## Introduction

As a consequence of the large increase in releases of radionuclides from mining and nuclear fuel cycle in the past century, considerable regulatory and public concerns have been raised on the impacts of elevated radioactivity on human and environmental health. Naturally occurring radioactive materials (NORM) and artificially produced radionuclides are the major contributors to environmental radioactivity and have been found in all types of ecosystems. In particular, radionuclides, such as long-lived ^137^Cs and ^90^Sr from Chernobyl^1^ and long-lived ^137^Cs and short-lived ^131^I from Fukushima,^2^ have been identified following these major nuclear accidents. The release of these radionuclides have significantly increased the level of ionizing radiation in contaminated areas and posed risks to human and wildlife.^2,3^ Although the annual doses at these hot spots such as the 30 km zone around Chernobyl reactor are considered declining after the accidents, high radiological concern with respect to long-term impacts from exposures to chronic low-dose-rate ionizing radiation remains.

An adverse outcome pathway (AOP) has been introduced as a conceptual framework to facilitate mechanistically based risk assessment. An important characteristic of the AOP framework is that it allows for utilization of information generated by cost-efficient New Approach Methodologies (NAMs), such as *in vitro* high-throughput screening (HTS), high-content (HT) OMICS, and *in silico* modeling to support regulatory decision making. Although the AOP framework is rapidly developing for chemical safety assessment, its application to nonchemical stressors such as radiation remains to be better developed. Establishment of the Radiation and Chemical (Rad/Chem) AOP joint topical group, a sub group of OECD’s Nuclear Energy Agency (NEA) High Level Group on Low Dose Research (HLG-LDR), is envisioned to help facilitate such AOP development in radiation research and foster broader implementation of AOPs into hazard and risk assessment.^4^ A number of ionizing radiation AOPs have subsequently been developed and submitted to the AOP repository AOPWiki (https://aopwiki.org/).

As a type of NAM, OMICS techniques (e.g., genomics, transcriptomics, proteomics, metabolomics, etc.) have been widely used to understand the toxic mechanisms of stressors and to develop novel biomarkers for environmental surveillance. Approaches to better utilize OMICS data to support hazard and risk assessment are also rapidly evolving in recent years. For instance, standardization of the OMICS data reporting has been proposed to meet regulatory requirements.^5−10^ Quantitative approaches such as benchmark dose (BMD) modeling have been adapted to the OMICS data for estimating points of departure (POD) that are relevant for setting safety thresholds. The PODs estimated by BMD modeling of bioassay data are useful for direct comparison (based on the same benchmark response/BMR level) of sensitivity across stressors and species,^11^ as well as for causation reasoning (i.e., dose and time concordance for sequentially occurring biological events) that is essential for identifying new AOPs and weight of evidence assessment (WoE) of the assembled AOPs.^12,13^ Among the POD approaches, the transcriptomic POD (tPOD) approach has been adopted in many studies and demonstrated to be a promising NAM to inform chemical screening and hazard identification.^11,14−20^ Using tPOD alone to inform hazard assessment, however, still has some limitations and uncertainties, as the information is only generated from a low (i.e., molecular) and single (i.e., gene expression) level of biological organization, which may not be sufficiently representative of complex physiological systems. The use of integrated OMICS analysis (multiomics) for POD estimation may improve the reliability of such approach. To date, only one study seems to have reported BMD modeling based on multiomics (transcriptomics and metabolomics) analysis; albeit, the tPOD and metabolomic POD (mPOD) were calculated separately without fully integrating multiple layers of understanding.^21^ Furthermore, there seems to be no study investigating how PODs derived from OMICS analyses can be used to support weight of evidence (WoE) assessment of AOPs.

Based on the previous advances and remaining research needs, the present study was conducted with the main aims of: (1) generating new empirical data using multiomics (transcriptomics and metabolomics) analysis and functional bioassays to better understand the effects of chronic low-dose ionizing radiation, using the model aquatic crustacean *Daphnia magna* and γ radiation as prototypes; (2) developing a biostatistical-bioinformatic workflow for multiomics integration and POD (moPOD) estimation; and (3) investigating how a combination of multiomics and functional PODs can support WoE considerations for an AOP network (AOPN) focusing on the effects of ionizing radiation. Transcriptomics and metabolomics were combined to form a multiomics approach in this study, as (1) transcriptomics has been widely used in eco(toxicology) and radioecology to indicate early stress responses (upstream events) and understand toxic mechanisms; (2) the tPOD has been demonstrated to be useful by several studies; (3) metabolomic responses are considered more representative of phenotypic changes (downstream stress responses at the molecular and cellular level) compared to other omic responses; and (4) data generated by these two types of OMICS analysis cover both signaling and metabolic pathways that provide a relatively more holistic picture of global stress responses to radiation exposure.

## Materials and Methods

### Exposure and Dosimetry

*Daphnia magna* (DHI strain) were cultured in M7 medium under favorable conditions (16 h light/8 h dark, 20 ± 1 °C, pH 8 ± 2, dissolved oxygen >8 mg/L, density 50 mL of medium per daphnid), as recommended by the OECD Test Guideline 211.^22^ The daphnids were fed with concentrated green algae *Raphidocelis subcapitata*, corresponding to 0.1 mg of total carbon per daphnid per day.^22^ The setup of γ radiation exposure was similar to that described elsewhere (Supporting Information (SI), Figure S1).^23^ Briefly, γ radiation was emitted from a ^60^Co (8 Ci) source at the FIGARO irradiation facility^24^ of Norwegian University of Life Sciences (NMBU, Ås, Norway). Six nominal dose rates (0.4, 1, 4, 10, 40, and 100 mGy/h) and a control group (background) were included in the tests. The dose rates reflected the distances between the exposure units and the source. The dose rates tested in this study were at a similar magnitude as dose rates measured immediately after serious nuclear events (e.g., Mayak Pa, Russia), thus representing a worst-case scenario for environmentally realistic exposures.^23^ Ten neonatal (<24 h old) daphnids placed in 40 mL of culture medium in a 50 mL plastic beaker functioned as one exposure unit (biological replicate). Landauer nanoDot and a Landauer microStar nanoDot reader (Landauer, Glenwood, IL), calibrated with certified calibration nanoDot, were used to measure actual exposure dose rates throughout the experiment, as previously described.^25^ The measurements included the inherent max and minimum dose rate intervals for each exposure unit. The total doses (mGy) received by the daphnids were estimated from the average dose rates to water (Dw), assuming random movement of the daphnids in the exposure unit, multiplied by the exposure time.

Due to space limitation, the radiation exposure was repeated three times to generate sufficient materials for different types of analysis: (1) after 4 and 8 days of exposure, three daphnids were pooled and stored in RNALater (Qiagen, Hilden, Germany) for transcriptomic analysis (*n* = 5). The two sampling time points were chosen, as at 4 days old, no ovulation had taken place in any of the daphnids, and at 8 days, all daphnids had the first batch of eggs but none had been released from the brood chamber. (2) After 8 days of exposure, 10 daphnids were frozen in liquid nitrogen for metabolomic analysis (*n* = 10). Samples were not collected after 4 days due to insufficient amount of materials for metabolomics. (3) after 8 days of exposure, individual daphnids were sampled (one daphnid per replicate) for functional bioassays, such as reactive oxygen species (ROS) production assay (*n* = 3), mitochondrial membrane potential (MMP) assay (*n* = 3), and whole-organism ATP pool (*n* = 3). Samples for OMICS and ATP determination were stored in −80 °C until use, whereas the remaining were used immediately for ROS and MMP assays. The remaining daphnids were stored as backup samples at −80 °C. An overview of the experimental setup can be found in the SI (Figure S1). pH and dissolved oxygen were measured before and after the exposure, as detailed in SI-1.

### Transcriptomic Analysis

The detailed procedure of transcriptomic analysis is described in the SI. Briefly, total RNA was extracted using an RNeasy Plus Mini kit (Qiagen, Hilde, Germany) and quality (260/280 > 1.8, yield >500 ng, and unique RNA peaks on gel with clear background) assured using a Nanodrop ND-1000 (Nanodrop Technologies, Wilminton, DE) and Agilent Bioanalyzer and RNA 6000 Nano chips (Agilent Technologies, Santa Clara, CA). RNA sequencing was performed by Beijing Genome Institute (BGI) using the BGISEQ-500 platform. The raw data (FASTQ files) have been submitted to the public repository database Gene Expression Omnibus (GEO, https://www.ncbi.nlm.nih.gov/geo/) with an accession number of GSE207246. Alignment of reads to the reference genome of *D. magna* (GenBank assembly accession: GCA_003990815.1) was performed using the OmicsBox software (BioBam Bioinformatics, Valencia, Spain). Functional annotation of the transcripts was conducted using the BLAST2GO function^26^ in OmicsBox. An ortholog mapping between *D. magna* and the fruit fly *Drosophila melanogaster* was performed using BLAST2GO to allow for utilization of advanced bioinformatics tools developed for *D. melanogaster*.

### Metabolomic Analysis

The detailed procedure of metabolomic analysis is described in the SI. Briefly, pooled Daphnia were delivered to Shanghai ProfLeader Biotech Co. (Shanghai, China) for untargeted metabolomic analysis using an Agilent 7890A gas chromatography system coupled to an Agilent 5975C inert MSD system (Agilent Technologies Inc.). The structural identification of differential metabolites was performed by applying the AMDIS software to deconvolute mass spectra from raw GC–MS data, and the purified mass spectra were automatically matched with an in-house standard library including retention time and mass spectra, Golm Metabolome Database, and Agilent Fiehn GC/MS Metabolomics RTL Library.

### Confirmative Bioassays

Several targeted bioassays were conducted as previously described^23,25^ to support the multiomics analysis. The detailed experimental procedures are described in the SI. Briefly, the measurement of cellular and mitochondrial reactive oxygen species (ROS) was conducted using the fluorescent probes, 2′,7′-dichlorodihydrofluorescein diacetate (H_2_DCFDA) and dihydrorhodamine 123 (DHR123, Thermo Fisher Scientific, Waltham, MA), respectively. The mitochondrial membrane potential was measured using the fluorescent probe tetramethylrhodamine methyl ester perchlorate (TMRM, Thermo Fisher, Waltham, MA, USA). The whole-organism ATP pool was quantified using a luminescent ATP detection assay kit (Abcam, Cambridge, UK). The bioassay results were normalized to the weight of individual *D. magna* calculated from the measured length according to the length–weight regression model proposed for this species.^27^ In addition, apical end points such as molting frequency (total number of molts) and growth (body length) were also measured.

### Benchmark Dose Modeling

**Figure 1:**
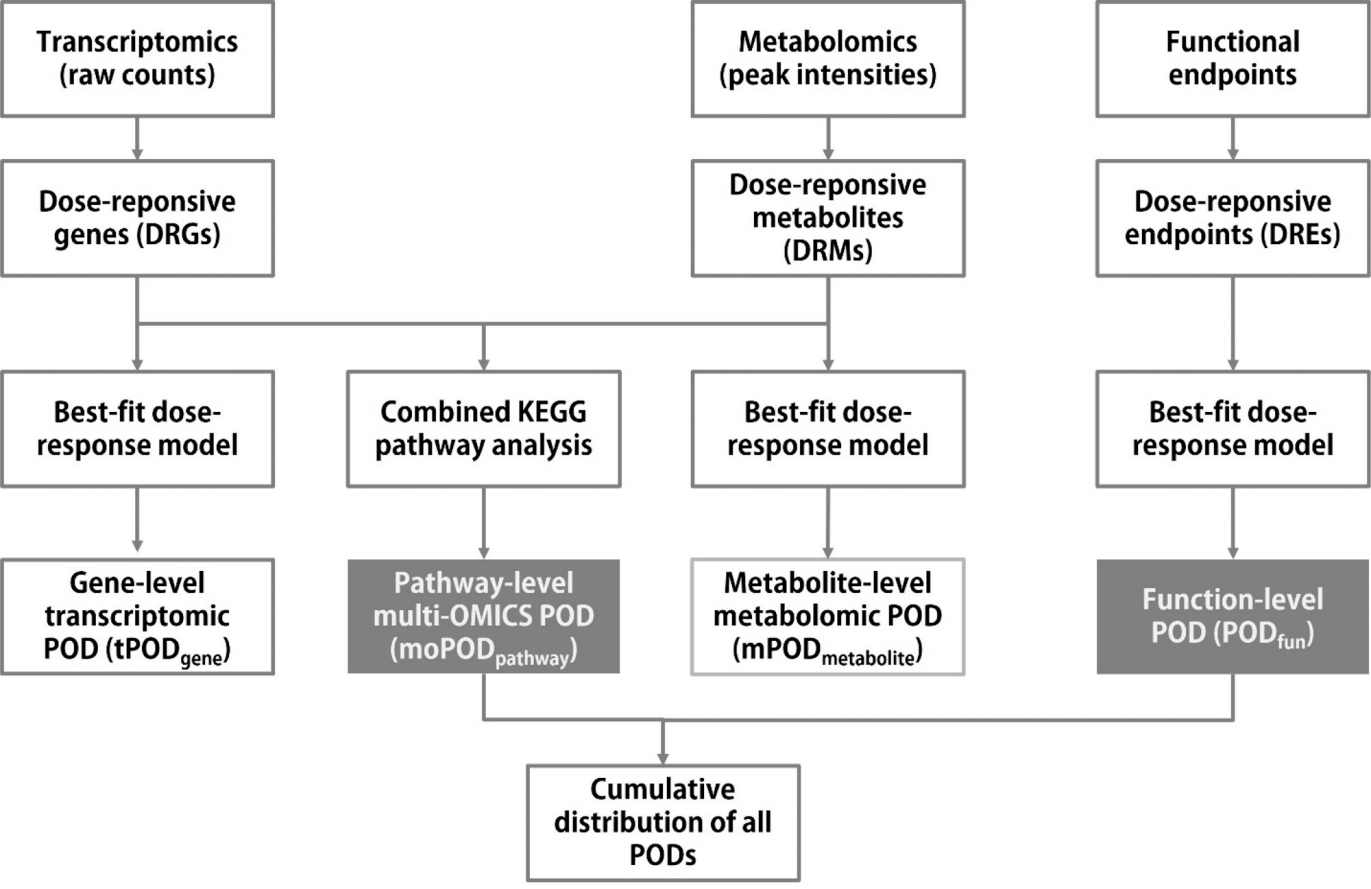
Workflow for multiomics integration and point of departure (POD) estimation.

Benchmark dose (BMD) modeling was performed using the DRomics package v2.4-0^28^ in the R v4.2.0 statistical environment.^29^ Briefly, both transcriptomic (4 and 8 d of exposure) and metabolomic data were log2 transformed. The significant dose-responsive genes (DRG) and metabolites (DRM) were identified using the quadratic method with a False Discovery Rate (FDR) less than 0.05. The DRGs and DRMs were fitted to predefined dose–response models, including linear, Hill, exponential, Gauss-probit, and log-Gauss-probit to identify the best-fit dose–response curves (monotonic increase, monotonic decrease, U-shaped, bell-shaped). The benchmark doses plus one standard deviation (BMD-1SD), corresponding to 10% benchmark response (BMR10), were derived from the dose–response curves, according to the guidance from the European Food Safety Authority (EFSA).^30^ The estimated BMDs were referred to as POD_molecule_ for transcriptomics (tPOD) or metabolomics (mPOD, Figure 1).

### Multiomics Integration

The DRGs and DRMs data were further integrated using MetaboAnalystR v5.0.^31^ A hypergeometric test (*p* < 0.05) was performed to identify combined KEGG pathways that were enriched by DRGs and DRMs (Figure 1).

### Pathway POD Estimation

The calculation of pathway-level POD was performed according to an established method, as described elsewhere.^19^ Briefly, the POD of a combined KEGG pathway was calculated based the geometric mean of the molecular-level PODs estimated for the supporting DRGs and DRMs. Both molecule- and pathway-level PODs were ranked by percentile and displayed in an empirical cumulative distribution function (ECDF) plot for visualization (Figure 1).

## Results and Discussion

### Exposure

The pH of the exposure media was 8.4 ± 0.3 and dissolved oxygen higher than 8.3 ± 0.2 mg/L throughout the exposure. The radiation dosimetry (Table S1) showed agreement between nominal and measured dose rates to water (Dw).

### Dose–response patterns

Results from the transcriptomic analysis showed that the majority of the DRGs were exposure duration specific, with only 80 genes in common between 4 and 8 days (Figure S2, and Table S2). The four major dose–response patterns were similarly distributed after 4 days of exposure, whereas more DRGs showed monotonic increase or decrease after 8 days (Figure S2, and Table S3). For DRM, a large proportion showed monotonic decreasing trend. The genes and metabolites displaying consistent dose–response patterns have great potentials to be further developed as biomarkers for hazard assessment of ionizing radiation as providing consistent causality between exposure and effects along the dose-rate–response relationship. The complete gene ontology (GO) and pathway analysis of DRGs and DRMs can be found in the SI (Tables S4–S8). Results from the functional assays showed increased cellular and mitochondrial ROS formation, whereas decreased MMP and whole-organism ATP pool were observed in a dose rate-dependent manner (Figure S3). These observations were in line with our previous findings using the same experimental setup.^23^ No significant effect on molting or growth was observed after 8 days (data not shown), which was also in agreement with our previous report.^23^ Nevertheless, it was expected that if the exposure prolonged, significant effect on reproductive capacity at a later life stage may occur, as evidenced by our previous work.^23^

### Combined pathway

**Figure 2:**
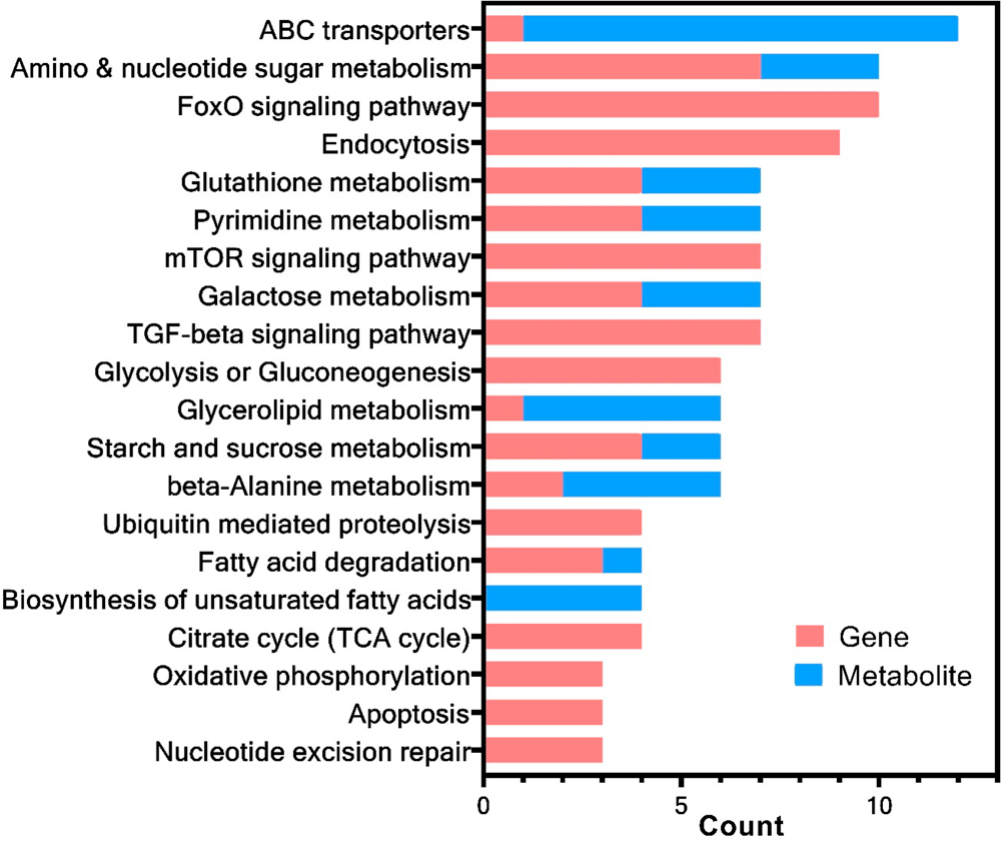
Combined KEGG pathways enriched by dose-responsive genes and metabolites.

The functional integration of dose-responsive multiomics data (Figure 2 and Table S9) showed that several KEGG pathways were highly relevant to the known toxic mechanisms of ionizing radiation, including oxidative stress, DNA damage, mitochondrial dysfunction, protein degradation, and apoptosis. Ionizing radiation is known to induce adverse effects at different levels of biological organization, from molecules to individuals, in several aquatic invertebrates (e.g., ref (32)). γ emitters can produce free radicals as an initial event through Compton effects and/or endogenous redox reactions^33,34^ that can result in direct effects at molecular and cellular levels. As expected, an increase in ROS level was observed in the present study, accompanied by an increase in glutathione metabolic pathway activity in response to induced oxidative stress. These results are consistent with those found in previous studies, where induction of cellular and mitochondrial ROS formation was observed in daphnids in response to the same ^60^Co source.^23,25^ Interestingly, no significant change in ROS level was found at the highest dose rate for both cellular and mitochondrial ROS, suggesting the triggering of antioxidant defense mechanisms at this dose rate, as previously reported.^25^ The induction of antioxidants in response to ROS formation has also been documented in other aquatic invertebrates (e.g., *Paracyclopina nana*(35) and *Tigriopus japonicus*(36)) exposed to ^137^Cs. Despite the tight regulation of these free radicals by endogenous antioxidants, excessive production of ROS can result in oxidative damage in macromolecules, such as lipids, proteins, and DNA at subcellular and cellular levels.^37^ In mitochondria, such oxidative damages may additionally suppress the energy production in respiration. γ radiation has been linked with mitochondrial disruption in daphnids after exposure to either ^60^Co^23,25^ or ^137^Cs,^38^ which is in accordance with the observed responses in glycolysis, TCA cycle, OXPHOS (and decreased MMP), and ATP pool (albeit not statistically significant), as well as the reduction in oxygen consumption during respiration. In addition to oxidative damage, ionizing radiation can directly cause double-strand breaks (DSBs) in DNA, which is associated with corresponding impacts on downstream events in the pathway leading to reproductive suppression. γ radiation-induced time and dose-dependent DNA damage has been well-documented in *D. magna*, as early as 2 days of exposure^25^ and across three successive generations,^39^ at dose rates as low as 0.007 mGy/h and up to 106 mGy/h. DNA damage associated with γ radiation (^137^Cs) has also been reported in *T. japonicus* after exposure to a dose rate of 1700 mGy/h^36^ and linked with decreased growth and impacted reproductive output. As a key downstream response, apoptosis can be triggered by both oxidative stress and DNA damage after exposure to radiation. The present study identified that apoptotic signaling was induced in *D. magna*, which again agrees with previous studies using similar dose rates (0.41–106 mGy/h).^25^ The role of oxidative stress and DNA damage in the regulation of apoptotic signaling has also been highlighted in fish (*Salmo salar*^40^ and *Danio rerio*(41)) after exposure to the same ^60^Co source as that used in this study. When occurring during oogenesis, apoptosis may result in the loss of oocytes and consequently inhibit reproduction. The alteration of oogenesis through apoptosis in response to γ radiation exposure has been well-documented in *Drosophila* after exposure to ^137^Cs at a total of 40 Gy.^42^ Even though the direct impact of γ radiation in the reproductive cycle of *D. magna* was not determined in this study, previous results have showed a correlation between ROS-induced DNA damage, impairment of oocyte development, and apoptosis-associated reproductive decline at the same dose rates.^23^

### Point of Departure

**Figure 3:**
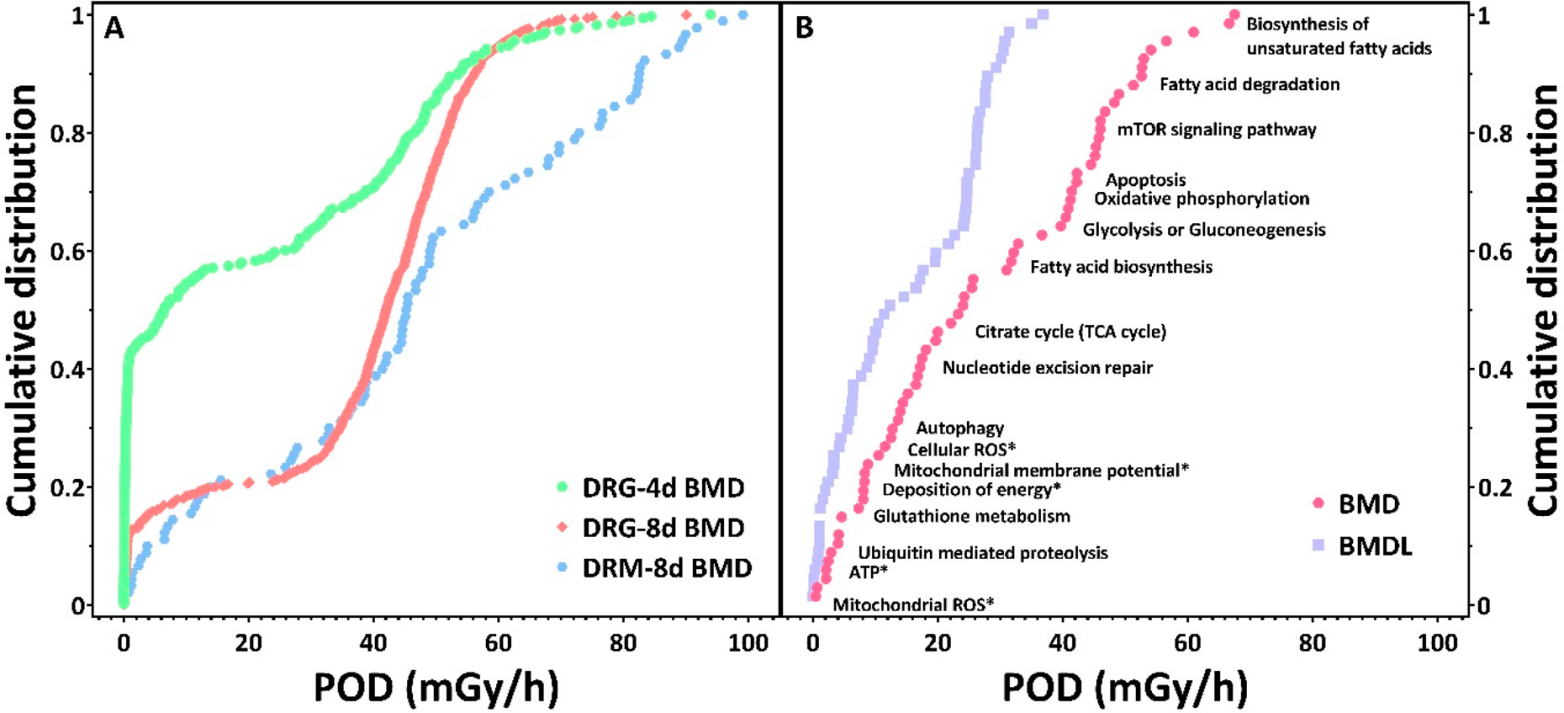
Empirical cumulative distribution function (ECDF) of (A) transcriptomic point of departure (tPOD) after 4 days (4 d) and 8 days (8 d) of exposure and metabolomic POD (mPOD) after 8 days. (B) Ranking and distribution of combined multiomics and function-level PODs. BMD, benchmark dose; BMDL, benchmark dose lower limit; SD, standard deviation.

The cumulative distribution of BMDs at transcriptional (tPOD) and metabolic (mPOD) pathways and functional levels (Figure 3) showed that oxidative damage related functions had relatively low BMDs, followed by DNA damage, energy metabolism, and apoptosis. These were in agreement with a previously proposed AOP network linking ROS production to reproduction decline.^23^

### Support for AOPN

**Figure 4:**
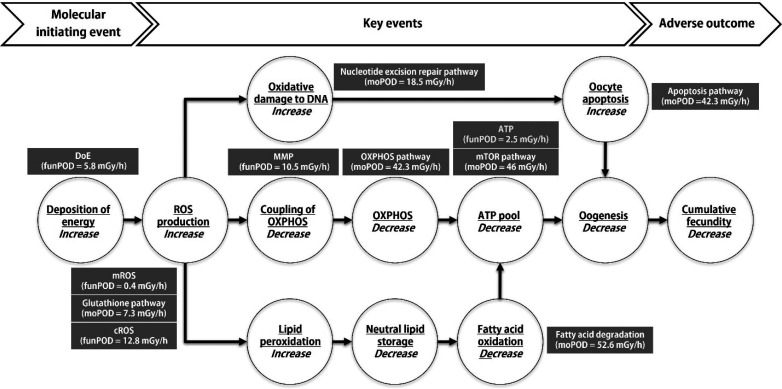
Mapping of points of departure (PODs) to the adverse outcome pathway network (AOPN) for ionizing radiation. funPOD, functional level POD; moPOD, multiomics POD; DoE, deposition of energy; ROS, reactive oxygen species; MMP, mitochondrial membrane potential; OXPHOS, oxidative phosphorylation.

The estimated PODs of the functional end points (funPOD) and the pathway-level multiomics PODs (moPOD) were overlaid with the key events in the AOP network (Figure 4). Genes and metabolites supporting the pathways can be found in the SI (Tables S2 and S3). Except for ATP, the enriched multiomics pathways, functional end points, and their PODs were well-aligned with several KEs in the AOP network, thus providing additional empirical evidence to support dose concordance of the AOPs. ATP did not follow such dose concordance, probably due to contribution by novel mechanisms associated with radiation-induced energy depletion that was not completely captured by the proposed AOP network. The temporal concordance was not assessed due to the great difference between the transcriptomic profiles after 4 and 8 days of exposure and the lack of metabolomics data for 4 days of exposure. Since temporal concordance is also an important element of the WoE considerations for AOPs, this aspect should be taken into account when designing future moPOD studies. As conceptual AOPs and AOPNs are often generated by narrative reviews to define relevant causal relationships, provide WoE assessment, and propose hypothesis to experimentally evaluate, broad-content analytical approaches such as RNA-sequencing and untargeted metabolomics are expected to provide confirmatory, contradictory, and explorative data. These multitude of quantitative data would be highly valuable for critical assessment of the WoE, to adjust and expand the knowledge domain or even develop new AOPs of relevance.

### Regulatory Relevance

Next generation risk assessment aims at supporting development of more mechanistically informed risk assessment and takes advantage of data spanning multiple levels of organization and assay formats. Inclusion of complementary data such as that generated by combined RNA sequencing and untargeted metabolomics and the associated biostatistical-bioinformatics workflow represents a set of novel NAMs to support AOP development through identifying PODs that collectively contribute to evaluate WoE, develop new AOPs, provide quantitative data for Integrated Approaches to Testing and Assessment (IATA), and identify thresholds for perturbations along the AOP continuum. Although such efforts have been actively pursued in the chemical research field,^43^ similar efforts are in their infancy within the radiation research and regulatory community.^4^ A lack of standardized and thoroughly evaluated AOPs for ionizing radiation has so far limited the use of OMICSs based POD in radiobiology, radioecology, and radiation protection. Thus, further work is needed to demonstrate the benefit of OMICSs based POD, especially related to chronic low-dose-exposure situations, before these technologies are incorporated in regulatory systems. The current paper specifically demonstrates how a suite of PODs for molecular, physiological, and phenotypic effects can pinpoint relevant toxicity mechanisms, aid identification of ecologically relevant AOP components to support laboratory to field extrapolations, and ultimately aid in defining environmental monitoring designs using more mechanistically informative bioassays. Such effort is not only essential for identification of the most sensitive toxicity end points but would also assist characterizing dose rates and doses where cascading events of AOPs and AOPNs are collectively expected to occur. Studies that demonstrate the use of AOP-informed PODs to characterize relative biological effectiveness of different radiation types and quality (e.g., α, β, γ, neutron radiation, etc.), quantification of tissue- and organ-specific differences in response, and deciphering the interspecies differences in radiosensitivity would be expected to improve future radiation protection assessments by reducing the overall uncertainty. Such efforts would be most useful for the development of mechanistically oriented hazard and risk assessment approaches both associated with ionizing radiation and with coexposure to other nonchemical and chemical stressors (i.e., multiple stressors).^44^

### Uncertainties and Limitations

Although the moPOD is considered a one-step-further approach compared to tPOD, its use is still affected by some unresolved uncertainty issues and limitations. First, the algorithm for calculating the pathway BMD/BMDL might not be optimal, as evidenced by the observation of smaller BMDs compared to BMDLs for a few combined pathways such as “ubiquitin mediated proteolysis” and “nucleotide excision repair”. This was likely due to the large variation in the BMD values and a lack of BMDLs for some supporting molecules. How to improve the algorithm for calculating pathway BMD may warrant further investigation. Second, the lack of annotations, especially for metabolites, still represents a bottleneck for maximizing the output of the moPOD approach. As shown in Figure 2, most of the multiomic pathways were dominated by supporting genes rather than a balanced distribution of genes and metabolites, possibly due to the exclusion of unannotated metabolites in the combined pathway analysis. This can only be resolved by constructing metabolomics databases capturing various species and standardization of metabolomics data reporting system, as proposed by Viant and co-workers.^45^ Third, although the moPODs can serve as additional evidence for the AOPs, they can only be used to support molecular/cellular level key events and relationships. Targeted bioassays measuring key events at higher levels of biological organization are still needed. The ultimate purpose of the current study is not to suggest the use of moPODs alone to support the AOPs but to include the moPOD approach in routine analysis and maximize the output of high-content OMICS to better understand (e.g., feedback and feedforward loops), support established AOPs (e.g., quantitative dose and temporal concordance support), and discover new AOPs.

## Supporting Information Available

Tables of measured dose rates of γ radiation, dose-responsive genes in *Daphnia magna* after 4 days of exposure to γ radiation, dose-responsive metabolites in *Daphnia magna* after 4 days of exposure to γ radiation, enriched gene ontology functions by dose-responsive genes in *Daphnia magna* after 4 and 8 days of exposure to γ radiation, enriched KEGG pathways by dose-responsive genes in *Daphnia magna* after 4 and 8 days of exposure to γ radiation, enriched KEGG pathways by dose-responsive metabolites in *Daphnia magna* after 8 days of exposure to γ radiation, and enriched KEGG pathways by a combination of dose-responsive genes and metabolites in Daphnia magna after 8 days of exposure to γ radiation and the estimated pathway-level points of departure (XLSX)
Discussions of materials and methods used and figures of overview of the radiation exposure setup and effect analyses, Venn diagram analysis of dose-responsive genes, and responses of cellular and mitochondrial reactive oxygen species, mitochondrial membrane potential, and ATP pool in *Daphnia magna* after 8 days exposure to γ radiation (PDF)
