# Supplementary material for: Multiomics Point of Departure (moPOD) Modeling Supports an Adverse Outcome Pathway Network for Ionizing Radiation
Source: Environ Sci Technol. 2023 Feb 17;57(8):3198–205. doi: 10.1021/acs.est.2c04917 (PMC9979642; doi:10.1021/acs.est.2c04917)
Supplement: Supplementary file 2 — es2c04917_si_002.pdf [file es2c04917_si_002.pdf]

Multiomics point of departure (moPOD) modeling supports an adverse outcome pathway network for ionizing radiation

You Song[*,†,‡], Keke Zheng[‡,§], Dag Anders Brede[‡,§], Tânia Gomes[†,‡], Li Xie[†,‡], Yetneberk Kassaye[‡,§], Brit Salbu[‡,§], Knut Erik Tollefsen[*,†,‡,§]

† Norwegian Institute for Water Research (NIVA), Økernveien 94, 0579 Oslo, Norway

‡ Centre for Environmental Radioactivity (CERAD), Norwegian University of Life Sciences (NMBU), Post box 5003, N-1432 Ås, Norway

§ Norwegian University of Life Sciences (NMBU), Faculty of Environmental Sciences and Natural Resource Management (MINA), Post box 5003, N-1432 Ås, Norway

Corresponding Authors

* You Song, Norwegian Institute for Water Research (NIVA), Økernveien 94, N-0579 Oslo, Norway, you.song@niva.no

* Knut Erik Tollefsen, Norwegian Institute for Water Research (NIVA), Økernveien 94, N-0579 Oslo, Norway, knut.erik.tollefsen@niva.no

SUMMARY OF SUPPORTING INFORMATION

Title Page: 1

Materials and Methods: Page S2 to S5

Supporting Figure S1: Page S6

Supporting Figure S2: Page S6

Supporting Figure S3: Page S7

Reference: Page S7

■ MATERIALS AND METHODS

pH and DO. The pH of the medium was measured before and after the exposure using WTW multi-parameter portable meter MultiLine® Multi 3420 coupled with WTW SenTix® 940 pH electrode with temperature sensor (Xylem Analytics, Weilheim, Germany). The same multi-parameter meter was also used to measure dissolved oxygen (DO) coupled with WTW-optical IDS dissolved oxygen sensor FDO® 925 (Xylem Analytics). Temperature, dissolved oxygen and pH were monitored throughout the exposure.

Transcriptomic analysis. ***RNA isolation***. Total RNA was extracted using the RNeasy Plus Mini kit (Qiagen) following the manufacturer's instructions. The purity and yield of the RNA samples were immediately assessed using a spectrophotometer (Nanodrop® ND-1000, Nanodrop Technologies, Wilminton, USA). The RNA integrity was checked using Agilent Bioanalyzer and RNA 6000 Nano chips (Agilent Technologies, Santa Clara, California, USA) according to the manufacturer's protocol. Intact RNA samples (clear peaks of RNA and flat bottom) with high purity (260/280>1.8) and sufficient yield (>500 ng) were stored at -80 ℃ until use.

RNA sequencing. The samples were submitted to Beijing Genome Institute (BGI) for RNA sequencing. The first step in the workflow involves purifying the poly-A containing mRNA molecules using poly-T oligo attached magnetic beads. Following purification, the mRNA is fragmented into small pieces using divalent cations under elevated temperature. The cleaved RNA fragments are copied into first strand cDNA using reverse transcriptase and random primers. This is followed by second strand cDNA synthesis using DNA Polymerase I and RNase H. These cDNA fragments then have the addition of a single 'A' base and subsequent ligation of the adapter. The products are then purified and enriched with PCR amplification. We then quantified the PCR yield by Qubit and pooled samples together to make a single strand DNA circle (ssDNA circle), which gave the final library. DNA nanoballs (DNBs) were generated with the ssDNA circle by rolling circle replication (RCR) to enlarge the fluorescent signals at the sequencing process. The DNBs were loaded into the patterned nanoarrays and pair-end reads of 100 bp were read through on the BGISEQ-500 platform for the following data

analysis study. For this step, the BGISEQ-500 platform combines the DNA nanoball-based nanoarrays and stepwise sequencing using Combinational Probe-Anchor Synthesis Sequencing Method.

Raw data processing. The raw sequencing data were pre-processed by BGI using the internal software SOAPnuke (BGI, https://github.com/BGI-flexlab/SOAPnuke) to filter reads, followed as: 1) Remove reads with adaptors; 2) Remove reads in which unknown bases(N) are more than 5%; 3) Remove low quality reads (defined as more than 20% of base with quality lower than 15 in a read). After filtering, the remaining reads are called "Clean Reads" and stored in FASTQ format. The FASTQ files have been submitted to the public repository database Gene Expression Omnibus (GEO, https://www.ncbi.nlm.nih.gov/geo/) with an accession number of GSE207246.

Alignment, annotation and ortholog mapping. Alignment of reads to the reference genome of *D. magna* (GenBank assembly accession: GCA_003990815.1) was performed using the OmicsBox software (BioBam Bioinformatics, Valencia, Spain). Functional annotation of the transcripts was conducted using the BLAST2GO function (Gotz et al., 2008) in OmicsBox. An ortholog mapping between *D. magna* and the fruit fly *Drosophila melanogaster* was performed to allow utilization of advanced bioinformatics tools developed for *D. melanogaster*.

Metabolomic analysis. ***Sample preparation***. Samples were delivered to Shanghai ProfLeader Biotech Co. (Shanghai, China) for untargeted metabolomic analysis. Pooled *D. magna* and 1350 μL of methanol/chloroform/water solvent (v/v/v=5:2:2, v/w=9, μL/mg) were added to a tube. The mixture was homogenized for 1 min in Tissuelyser JX-24 (Shanghai Jingxin Industrial Development Co., Ltd, China). The mixture was placed at -20 °C for 24 h prior to centrifugation at 16000 g and 4 °C for 15 min. Then, 1080 μL of supernatant was transferred into a new tube. The procedure was then repeated by adding 1080 μL of methanol. Another 540 μL of supernatant was transferred and combined with the first extraction. Then, 180 μL of mixture was added to a GC vial, containing 10 μL of internal standards (0.1 mg/mL of $^{13}C_3$-^{15}N-L-alanine, $^{13}C_5$-^{15}N-L-valine, $^{13}C_6$-^{15}N-L-leucine and $^{13}C_6$-^{15}N-L-isoleucine). The mixture was dried under gentle nitrogen stream. The vial with dry residue was added with 30 μL of 20 mg/mL methoxyamine hydrochloride in pyridine. The resultant mixture was vortex-mixed vigorously for 30 s and incubated at 37 °C for 90 min. A 30 μL of BSTFA (with 1% TMCS) was added into the mixture and derivatized at 70 °C for 60 min prior to performing GC-MS metabolomics analysis.

GC-MS analysis. Metabolomics instrumental analysis was performed using an Agilent 7890A gas chromatography system coupled to an Agilent 5975C inert MSD system (Agilent Technologies Inc., CA, USA). A HP-5ms fused-silica capillary column (30 m × 0.25 mm × 0.25μm; Agilent J&W Scientific, Folsom, CA) was utilized to separate the derivatives. Helium (>99.999%) was used as a carrier gas at a constant flow rate of 1 mL/min through the column. Injection volume was 1 μL in split mode with a ratio of 2:1, and the solvent delay time was 6 min. The initial oven temperature was held at 70 °C for 2 min, ramped to 160 °C at a rate of 6 °C/min, to 240 °C at a rate of 10 °C/min, to 300 °C at a rate of 20 °C/min, and finally held at 300 °C for 6 min. The temperatures of injector, transfer line, and electron impact ion source were set to 250 °C, 290 °C, and 230 °C, respectively. The impact energy was 70 eV, and data was collected in a full scan mode (m/z 50-600).

Raw data processing. The peak picking, alignment, deconvolution, and further processing of raw GC-MS data were referred to the previous published protocols (Gao et al. 2010). The final data was exported as a peak table file, including observations (sample name), variables (rt_mz), and peak intensity. The data was normalized against total peak intensities before performing univariate and multivariate statistics. The peak table file was imported to SIMCA (version 13.0, Umetrics AB, Umeå, Sweden), where multivariate statistical analysis, such as PCA and PLS-DA, were performed. All data were mean-centered and unit variance (UV)-scaled prior to multivariate statistical analysis. The quality of the models is described by the R2X or R2Y and Q2 values. R2X (PCA) or R2Y (PLS-DA) is defined as the proportion of variance in the data explained by the models and indicates the goodness of fit. Q2 is defined as the proportion of variance in the data predictable by the model and indicates the predictability of current model, calculated by cross-validation procedure.

Identification and structural validation of differential metabolites. The differential metabolites were determined by the combination of the Variable Importance in the Projection (VIP) value (>1) of PLS-DA model and the p values (<0.05) from two-tailed Student's t-test on the normalized peak intensities. Fold change was calculated as binary logarithm of average normalized peak intensity ratio between each radiation dose treatment and the control. The structural identification of differential metabolites was performed by applying the AMDIS software to deconvolute mass spectra from raw GC-MS data, and the purified mass spectra were automatically matched with an in-house standard library including retention time and mass spectra, Golm Metabolome Database, and Agilent Fiehn GC/MS Metabolomics RTL Library.

Confirmative bioassays. ***ROS assays***. Two fluorescent ROS probes, 2',7'-dichlorodihydrofluorescein diacetate (H_2DCFDA) and dihydrorhodamine 123 (DHR123) purchased from Thermo Fisher Scientific were used to measure cellular and mitochondrial ROS production in *D. magna*, respectively. Stock solutions of the probes (5 mM) were prepared in dimethyl sulfide (DMSO, *in vitro* grade, Sigma-Aldrich) and stored at -20 °C in the dark until use. Prior to analysis, the ROS probes were diluted in the culture (M7) medium as working solutions. The ROS assays (n=3) were performed as previously described. (Gomes et al., 2018) Briefly, individual daphnids were placed in 200 μL M7 medium containing 5 μM probe in separate wells of a 96-well black microplate (Corning Costar, Cambridge, MA, USA). The plate was incubated for 1 hour at room temperature in the dark. After incubation, the daphnids were washed three times with clean culture medium to remove excessive probes in the wells. The plates were immediately scanned using a VICTOR 3 microplate reader (PerkinElmer, Waltham, USA) with excitation/emission wavelengths of 485/538 nm. The results were normalized to the weight of individual *D. magna* calculated from the measured length according to the length-weight regression model proposed for this species. (Cauchie et al., 2000)

TMRM assay. As an indicator of oxidative phosphorylation (OXPHOS) coupling, MMP was measured using the fluorescent probe tetramethylrhodamine methyl ester perchlorate (TMRM, Thermo Fisher Scientific), as previously described. (Song et al., 2020) Stock solutions (5 mM) were prepared in DMSO and stored at -20 °C in the dark until further use. Shortly before analysis, the stock solution was diluted in M7 medium to make a final working concentration of 2 μM. Individual daphnids (n=3) were incubated in 200 μL TMRM working solution in separate wells of a 96-well black microplate (Corning Costar) at room temperature in the dark for 1h. After incubation, the animals were washed three times with M7 medium to eliminate excess TMRM in the assay solutions. The plate was immediately scanned using a VICTOR 3 microplate reader (PerkinElmer) with excitation/emission wavelength of 530/590 nm. The results were normalized to the calculated weight of *D. magna*.

ATP assay. The whole-organism ATP pool was quantified using the Luminescent ATP Detection Assay Kit (Abcam) following the manufacturer's protocol with minor modifications, as previously described. (Song et al., 2020) Briefly, pooled (2 individuals) D. magna (n=3) were homogenized in 225 μL lysis buffer (75 μL detergent + 150 μL ddH2O) using a Precellys orbital shaker bead mill (Bertin). The homogenate was centrifuged at 13,000 g (4°C) for 5 min. The supernatant (200 μL) was carefully transferred to a new tube. The ATP standards (0.00064, 0.0032, 0.016, 0.08, 0.4, 2, 10 μM) were prepared by diluting an ATP stock solution (10 mM)

in the ATP assay buffer to generate a standard curve. Each sample or standard (150 µL) was mixed with 50 µL substrate solution in a 96-well black microplate (Corning Costar). The plate was shaken (600-700 rpm) for 5 min using an orbital shaker and incubated in the dark for another 10 min. The luminescence was immediately measured using a MicroBeta2 microplate counter (PerkinElmer). The total ATP content (nmol) in each sample was calculated based on the standard curve and further normalized to the calculated weight of *D. magna*.

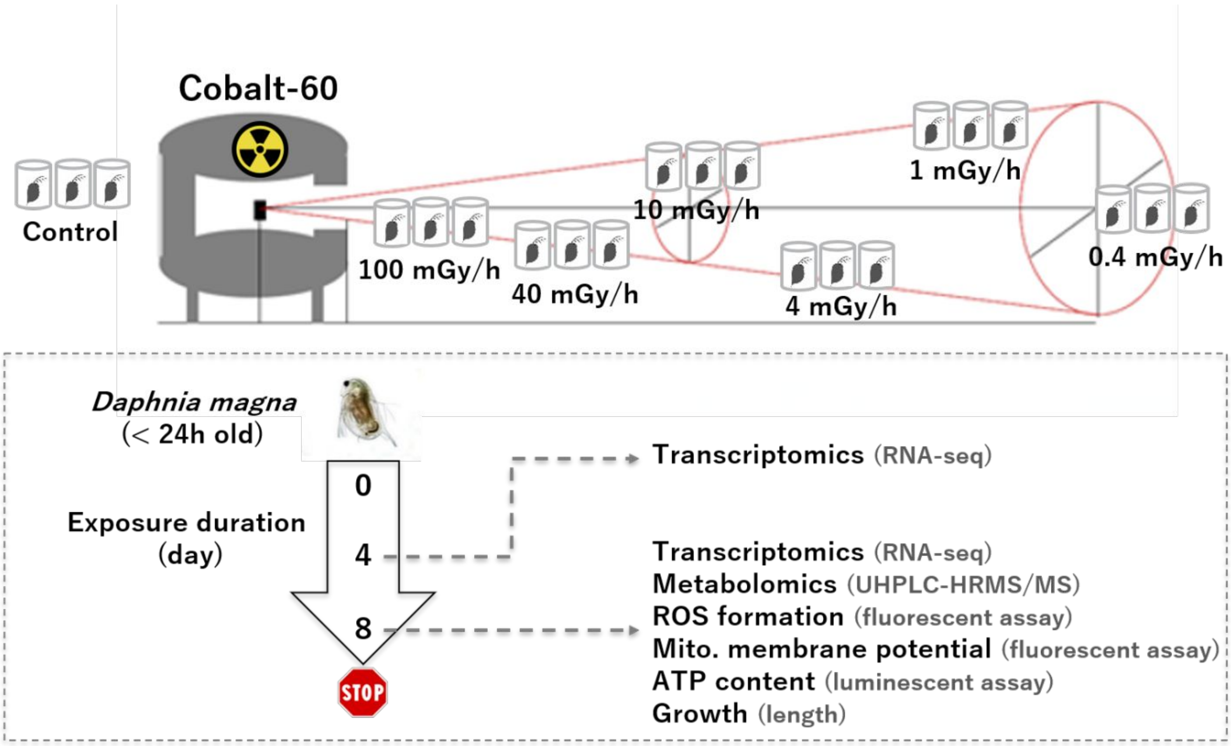

Figure S1. An overview of the radiation exposure setup and effect analyses.

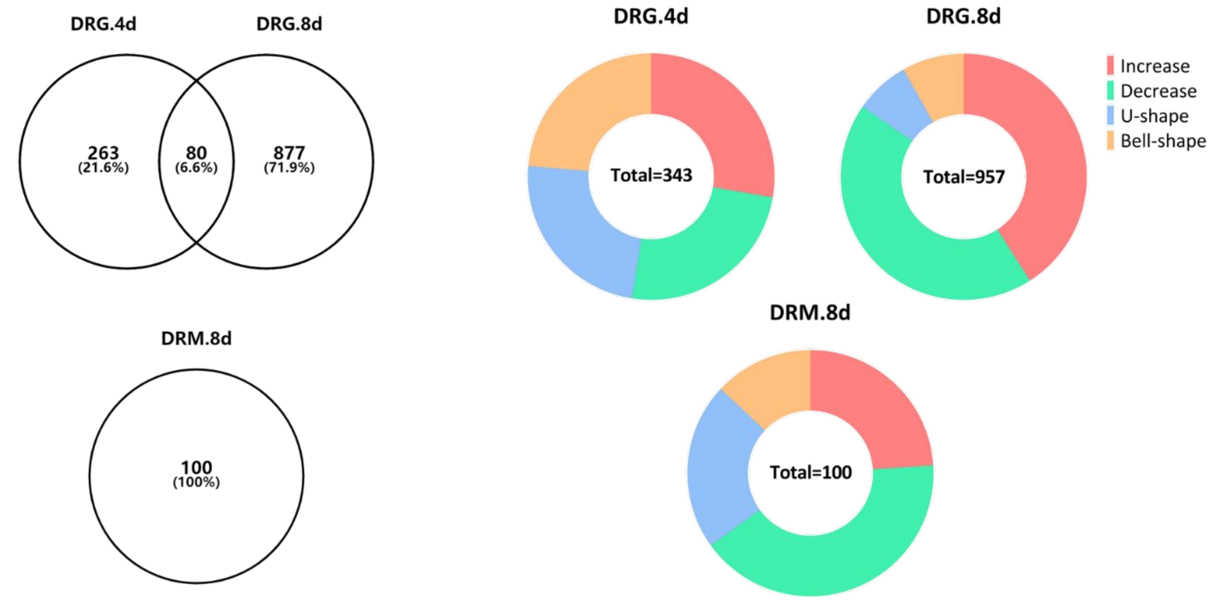

Figure S2. Venn diagram analysis of dose-responsive genes (DRG) after 4 days and 8 days exposure, and metabolites (DRMs) after 8 days exposure.

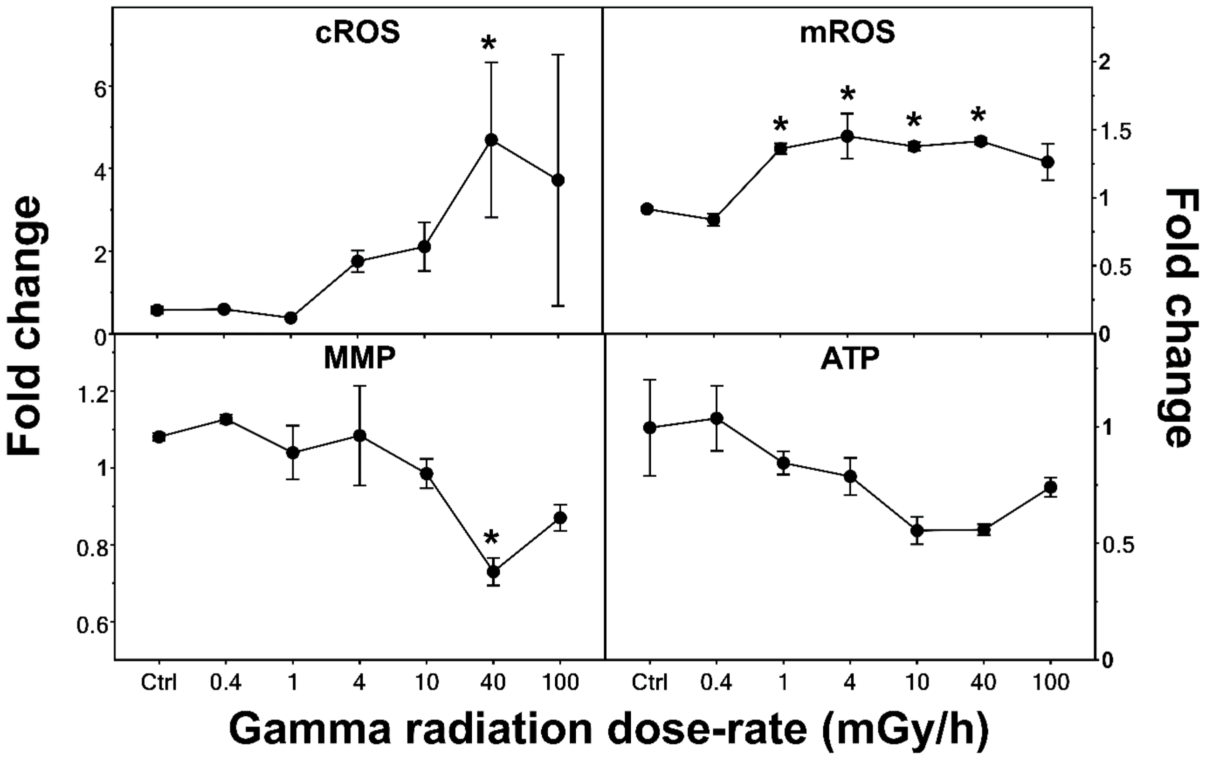

Figure S3. Responses of cellular (cROS) and mitochondrial reactive oxygen species (mROS), mitochondrial membrane potential (MMP) and ATP pool in *Daphnia magna* after 8 days exposure to gamma radiation.

References

Cauchie, H. M.; Thys, I.; Hoffmann, L.; Thome, J. P., In situ versus laboratory estimations of length-weight regression and growth rate of *Daphnia magna* (Branchiopoda, Anomopoda) from an aerated waste stabilization pond. *Hydrobiologia* **2000,** *421*, 47-59.

Gomes, T.; Song, Y.; Brede, D. A.; Xie, L.; Gutzkow, K. B.; Salbu, B.; Tollefsen, K. E., Gamma radiation induces dose-dependent oxidative stress and transcriptional alterations in the freshwater crustacean *Daphnia magna*. *Sci Total Environ* **2018,** *628-629*, 206-216.

Gotz, S.; Garcia-Gomez, J. M.; Terol, J.; Williams, T. D.; Nagaraj, S. H.; Nueda, M. J.; Robles, M.; Talon, M.; Dopazo, J.; Conesa, A., High-throughput functional annotation and data mining with the Blast2GO suite. *Nucleic Acids Res* **2008,** *36*, (10), 3420-35.

OECD, OECD guideline for the testing of chemicals. In *Daphnia magna reproduction test*, OECD: 2012; Vol. 211.

Song, Y.; Xie, L.; Lee, Y.; Brede, D. A.; Lyne, F.; Kassaye, Y.; Thaulow, J.; Caldwell, G.; Salbu, B.; Tollefsen, K. E., Integrative assessment of low-dose gamma radiation effects on *Daphnia magna* reproduction: Toxicity pathway assembly and AOP development. *Sci Total Environ* **2020,** *705*, 135912.

Lind, O. C.; Helen Oughton, D.; Salbu, B., The NMBU FIGARO low dose irradiation facility. *Int J Radiat Biol* **2019,** *95*, (1), 76-81.
